# Supplementary material for: Mapping network structures and dynamics of decentralised cryptocurrencies: The evolution of Bitcoin (2009–2023)
Source: PLoS One. 2025 Aug 18;20(8):e0329617. doi: 10.1371/journal.pone.0329617 (PMC12360650; doi:10.1371/journal.pone.0329617)
Supplement: Attachment — Submitted filename: response_to_reviewers.docx [file pone.0329617.s001.docx]

**Rebuttal letter, response to the editor and reviewers**

**Reviewer #1
Minor modifications recommended:**

- *Discussion of implications: The results are relevant to current debates on decentralization and regulation of cryptocurrencies. The discussion could be strengthened by a more critical reflection on these implications, especially in light of the endogenous concentration of power found.*

**Author**: Thank you for this suggestion. We have added a more comprehensive reflection on the implications of the endogenous concentration of wealth and power.

- *Limitations section: The limitations of the paper (e.g., use of annual cross-sections, omission of subtle temporal fluctuations) merit their own clearer section.*

**Author**: Thank you for this suggestion. We have now elaborated on the study limitations and explored future developments.

- *Readability to a broader audience: PLOS ONE has a wide readership. Some terms, such as "transitivity index" and "weakly vs. strongly connected components", would benefit from a brief explanation.*

**Author**: Good point. We have accompanied technical terms with plain language explanations.

- *Visualizations: Several figures (especially Figs. 1-2 and Figs. 5-6) deserve to be accompanied by more prominent captions or legends to be interpreted independently outside the main text.*

**Author**: Good point. We have extended and integrated the captions, as suggested.

- *Emphasis on originality: Although the paper refers to relevant literature (e.g., Kondor, Maesa), I recommend that it be made even more explicit what makes this paper different and pushes the boundaries of previous research.*

**Author**: Thank you for this remark. We have expanded on the relevance of our study in the initial section. We highlighted that our contribution consists of three main points: first, we have updated previous studies, which were limited to a few years of observation; second, we have offered a new categorisation of the Bitcoin’s life phases by capturing internal dynamics and external evolution (both points have been ignored in previous research); third, we provided a broad range of measures to disentangle the complexity of the system, while previous research tended to focus on specific aspects only (e.g., power law degree distributions). Furthermore, we tested for the rich-get-richer mechanism to include post-2015 data and we focused on centralisation, node persistence and wealth inequality. These points have been better specified in the revised version of the text.

**Reviewer #2**

**Minor modifications recommended:**

- *The manuscript is well-written, logically organized, and accessible to both technical and interdisciplinary audiences. While the introduction includes some speculative references to political events (e.g., US presidential election), these do not undermine the scientific integrity of the analysis. Still, the authors may consider rephrasing these in a more neutral tone.*

**Author**: Good point. However, there was no political intention behind as we simply tried to give context and ground our topic in the actual political scenario. But you are right, in these difficult times, better not to convey any political message and do an extra-effort to be as neutral as possible.

- *Add a brief discussion of limitations regarding address-level analysis (e.g., address reuse, clustering heuristics).*

**Author**: Good point. Following also a similar request by Reviewer 1, we have expanded on the study limits and included also this point.

- *Consider a diagram or figure to visualize the data pipeline from raw blockchain data to network construction.*

**Author**: Good point. We have added a diagram to visualise the data pipeline.

- *Tidy up punctuation around compound terms and ensure consistent hyphenation and spacing (e.g., “Exploration, Adaptation and Maturity” - “Exploration, Adaptation, and Maturity”).*
- *Clarify briefly how some of the early richest nodes were de-anonymized despite limited historic metadata.*

**Author**: Thank you for your suggestions. We have added clarifications to explain the point. Most of the deanonymisation techniques consisted of web scraping and searching for digital traces (e.g., some users publicly state their address in forums). Nonetheless, we acknowledge that only a small portion of addresses has been de-anonymised, almost only exchanges and big players. As clarified in the revised version of the text, we relied on previous works and Arkham Intelligence and BitInfoCharts.
